# Application of stochastic phenomenological modelling to cell-to-cell and beat-to-beat electrophysiological variability in cardiac tissue

**DOI:** 10.1016/j.jtbi.2014.10.029

**Published:** 2015-01-21

**Authors:** John Walmsley, Gary R. Mirams, Joe Pitt-Francis, Blanca Rodriguez, Kevin Burrage

**Affiliations:** a Department of Computer Science, University of Oxford, Oxford, United Kingdom; b School of Mathematical Sciences, Queensland University of Technology, Brisbane, Queensland, Australia

**Keywords:** Cardiac electrophysiology, Inter-cellular coupling, Stochastic simulation

## Abstract

Variability in the action potential of isolated myocytes and tissue samples is observed in experimental studies. Variability is manifested as both differences in the action potential (AP) morphology between cells (extrinsic variability), and also ‘intrinsic’ or beat-to-beat variability of repolarization (BVR) in the AP duration of each cell. We studied the relative contributions of experimentally recorded intrinsic and extrinsic variability to dispersion of repolarization in tissue. We developed four cell-specific parameterizations of a phenomenological stochastic differential equation AP model exhibiting intrinsic variability using APs recorded from isolated guinea pig ventricular myocytes exhibiting BVR. We performed simulations in tissue using the four different model parameterizations in the presence and the absence of both intrinsic and extrinsic variability. We altered the coupling of the tissue to determine how inter-cellular coupling affected the dispersion of the AP duration in tissue. Both intrinsic and extrinsic variability were gradually revealed by reduction of tissue coupling. However, the recorded extrinsic variability between individual myocytes produced a greater degree of dispersion in repolarization in tissue than the intrinsic variability of each myocyte.

## Highlights

• We modelled inter-cell and beat-to-beat repolarization variability in cardiomyocytes.
• We coupled the cells together into cardiac tissue.
• Reducing tissue coupling increased repolarization dispersion in tissue.
• Inter-cell variability had a greater effect on repolarization dispersion.

## 1 Introduction

Dispersion of repolarization is defined as a spatial heterogeneity in the time of repolarization between different regions of cardiac tissue. Dispersion of repolarization is associated with arrhythmic risk (Chauhan et al., 2006). A potential source of dispersion of repolarization is variability in action potential (AP) morphology and duration in the myocytes within cardiac tissue. Variability in the AP duration (APD) of cardiac myocytes may arise from naturally occurring differences in ion channel density between cells, the spatial location of the cell within the heart, and circadian rhythms (Jeyaraj et al., 2012). Cell isolation procedures may also affect the AP of cells that have been isolated from the myocardium (Yue et al., 1996). We describe these differences in AP morphology between different myocytes as ‘extrinsic variability’.

An alternative source of variability in APD is ‘intrinsic’ beat-to-beat variability of repolarization (BVR) in the same cell. Beat-to-beat variability in the APD of isolated cells (Zaniboni et al., 2000), beat-to-beat dispersion of repolarization in tissue (Hondeghem et al., 2001), and variability in the QT-interval at the ECG level (Thomsen et al., 2004) have all been reported. At a cellular level, BVR is an apparently random variation in the APD (Johnson et al., 2010). This is distinct from alternans, which is a regular short-long oscillation in the APD between subsequent beats. BVR at both a cellular and a whole-heart level has been shown to be a predictor of arrhythmogenic risk, although the mechanisms by which this occurs are unknown (Kääb et al., 2003, Johnson et al., 2010).

Extrinsic variability between different cell and tissue samples is difficult to reproduce experimentally, as it is specific to the sample under investigation. Computational modelling studies may provide a method to understand how this extrinsic variability contributes to dispersion of repolarization in the ventricles. Many simulation studies have focussed on heterogeneities that exist within the ventricle; for example transmurally, or in an apex-base direction (Efimov et al., 1996, Viswanathan et al., 1999, Keller et al., 2012). The first approaches to modelling consequences of variability in ion channel expression in single cells are now beginning to appear (Sarkar and Sobie, 2011, Walmsley et al., 2013, Britton et al., 2013, Gemmell et al., 2014).

Intrinsic beat-to-beat variability at a cellular level has also been suggested as contributing to dispersion of repolarization in tissue. Simulation studies by Pueyo et al. (2011), Lemay et al. (2011), and Heijman et al. (2013) demonstrated that the BVR in isolated cells induced by stochasticity in ion channel gating is dramatically reduced when cells are electrotonically coupled in tissue. These studies all showed that BVR can re-appear as the conductivity of the tissue decreases.

In this study, we investigate how dispersion of repolarization in the ventricular myocardium is affected by the relative contributions of intrinsic beat-to-beat variability, and extrinsic variability between different cells. We also investigate how these contributions are influenced by inter-cellular coupling, as motivated by the findings of Lesh et al. (1989), Lemay et al. (2011) and Pueyo et al. (2011), who observed that the degree of coupling between cells affected dispersion of repolarization.

Our simulations of intrinsic and extrinsic variability are based on experimental recordings from isolated guinea pig ventricular myocytes that exhibit both intrinsic and extrinsic variability. We use a phenomenological model that reproduces both cell-to-cell (extrinsic) and beat-to-beat (intrinsic) variability (Bueno-Orovio et al., 2008, Walmsley et al., 2010). We use four parameter sets that reproduce four specific isolated cell samples so that our simulations are based on physiological levels of single cell variability (Walmsley et al., 2010). We then couple these cells into a simulated tissue using a monodomain approach.

## 2 Methods

### 2.1 Reproducing intrinsic beat-to-beat variability

#### 2.1.1 Action potential model

Our phenomenological model of beat-to-beat intrinsic variability is based on the Bueno-Orovio–Cherry–Fenton (BOCF) phenomenological model, described in detail in Appendix A (Bueno-Orovio et al., 2008). We modified the BOCF AP model to incorporate intrinsic beat-to-beat variability as described by Walmsley et al. (2010). A Weiner process is a stochastic process whose increment *dW*_*t*_ on the interval [t,t+Δt] has mean 0 and variance Δt. We modified Eqs. (A.2), (A.3), (A.4) by including a Weiner increment in each, forming a system of stochastic differential equations (SDEs):

(1) $$\mathrm{dv}=((1-H(u-\theta_{v}))(v_{\infty }-v)/\tau_{v}^{-}-H(u-\theta_{v})v/\tau_{v}^{+})\;\mathrm{dt}+\sigma_{v}\;{\mathrm{dW}}_{t}^{v},$$

(2) $$\mathrm{dw}=((1-H(u-\theta_{w}))(w_{\infty }-v)/\tau_{w}^{-}-H(u-\theta_{w})w/\tau_{w}^{+})\;\mathrm{dt}+\sigma_{w}\;{\mathrm{dW}}_{t}^{w},$$

(3) $$\mathrm{ds}=(((1+\tanh(k_{s}(u-u_{s})))/2-s)/\tau_{s})\;\mathrm{dt}+\sigma_{s}\;{\mathrm{dW}}_{t}^{s},$$

where *σ*_*v*_, *σ*_*w*_, and *σ*_*s*_ are constants changing the magnitude of the Weiner increment (see, for example, Kloeden and Platen, 2011). The superscript denotes an independent Weiner process for each variable.

#### 2.1.2 Reproduction of experimentally observed variability

**Fig. 1:**
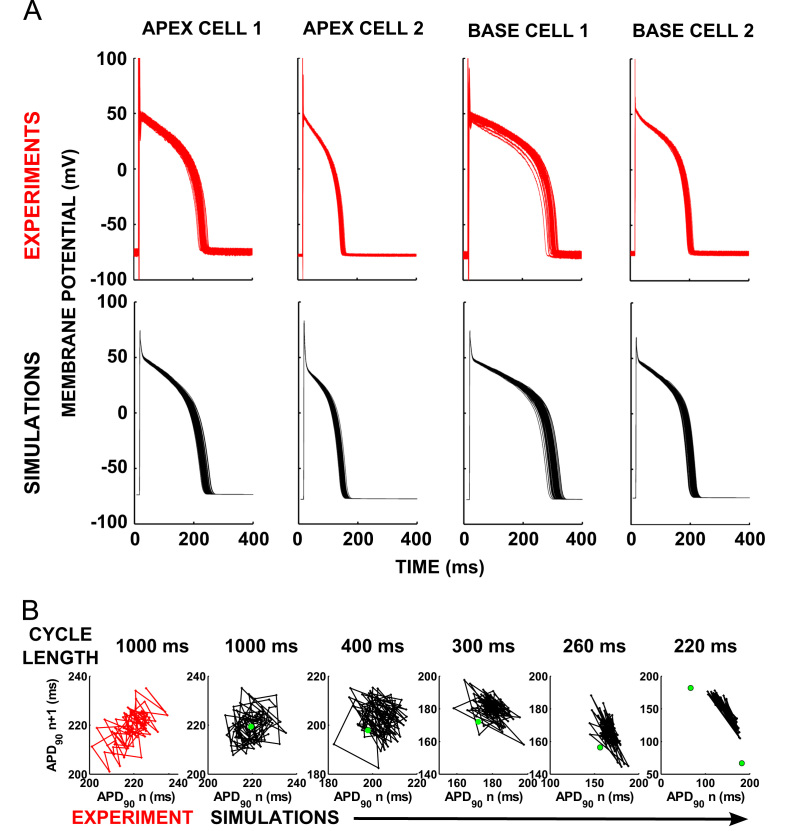
Experimental and simulated temporal variability in repolarization. (A) Experimental data showing repeated 1 Hz stimulations of isolated guinea pig ventricular myocytes from the apex and the base of the heart. Simulated reproductions of the data using four parameterizations of the SDE model are shown below each set of experimental data. Modified from Walmsley et al. (2010). (B) Poincaré plots of the original experimental data (red) and those generated from the guinea pig apex cell 1 parameter set are shown at pacing cycle lengths of 1000 ms, 400 ms, 300 ms, 260 ms, and 220 ms for the stochastic (black) and deterministic (green dot) versions of the model. (For interpretation of the references to colour in this figure caption, the reader is referred to the web version of this paper.)

Examples of APs simulated using the parameter sets for the SDE model derived by Walmsley et al. (2010) for each of the four cells are shown in Fig. 1A (bottom row). These simulations show good qualitative and quantitative agreement with both the experimentally recorded action potential morphologies, and spread of repolarization times shown in Fig. 1A (top row). There was no variation in the resting potential in the simulations. Fitting of the noise terms was successful in matching the mean and variance of the experimental data (Table 1). APD in the simulations and experiments was quantified by the time from upstroke to 90% repolarization (APD_90_). Poincaré plots demonstrate the degree of temporal variability by plotting the APD_90_ of each beat against the APD_90_ of the preceding beat. Fig. 1B shows Poincaré plots of the temporal variability in APD for apex cell 1 from the experimental data and simulations. The fitted noise terms from Walmsley et al. (2010) are shown in Table 1. For each parameter set we therefore have the stochastic version as shown in Fig. 1, and also a deterministic version where each noise term is set to zero.

**Table 1:** Experimental and simulated variability in APD_90_.

Parameter	Apex 1	Apex 2	Base 1	Base 2
Mean (exp) (ms)	219.5	133.7	286.5	185.8
Variance (exp)	37.69	16.80	75.56	21.88
*σ*_*v*_	0	0	0	0
*σ*_*w*_	0.0012	0.0015	0.0011	0.0011
*σ*_*s*_	0.0012	0.002	0.0013	0.0012
Mean (sim) (ms)	223.5	127.3	268.7	182.3
Variance (sim)	39.22	17.64	77.97	23.73

### 2.2 Two-dimensional tissue simulations

#### 2.2.1 Modelling conduction in tissue

All simulations were performed in a two-dimensional tissue sheet of dimension 1 cm ×1 cm. Inter-cellular coupling was simulated using a monodomain approach, where Eq. (A.1) was spatially extended to

(4) $$\frac{\partial u}{\partial t}=\nabla \cdot (D\;\nabla u)-(J_{\mathrm{fi}}+J_{\mathrm{so}}+J_{\mathrm{si}}-J_{\mathrm{stim}}).$$

We applied zero-flux Neumann boundary conditions to the tissue. In all simulations, the stimulus was applied at the left-hand edge of the tissue (x<0.03cm).

We used four different values of the diffusion coefficient *D* to investigate the effects of reduced coupling upon temporal variability of repolarization. In the original BOCF model *D*=1.171 cm^2^ s^−1^ was used for tissue simulations, based on experimental measurements of human ventricular cell dimensions and cytoplasmic resistivity (Bueno-Orovio et al., 2008), giving a conduction velocity of 72 cm s^−1^. Simulations were also performed at 50%, 10%, and 5% of this value (*D*=0.586 cm^2^ s^−1^, 0.117 cm^2^ s^−1^, and 0.059 cm^2^ s^−1^, respectively), giving conduction velocities of 51 cm s^−1^, 23 cm s^−1^ and 16 cm s^−1^, respectively.

#### 2.2.2 Determining the effect of intrinsic variability alone

To investigate the effect of intrinsic BVR alone without extrinsic variability between cells, we created a sheet of tissue with a homogeneous AP model. We repeated this for each of the four parameter sets in Section 2.1, giving four different sheets of tissue. 50 stimuli were applied at a pacing cycle length of 1000 ms. We repeated the simulations for both the deterministic version and the stochastic versions of the AP model to observe the effects of intrinsic variability alone. In order to ensure that simulations using the stochastic AP models were comparable, the same pseudo-random number sequence was used for each stochastic simulation. A different sequence was generated for each node in the computational mesh.

#### 2.2.3 Effect of fast pacing rates on dispersion of repolarization

We also investigated the consequences of the alternans-like behaviour induced in the stochastic AP model at short cycle lengths shown in Fig. 1B. In order to determine whether this alternans affected the magnitude of dispersion of APD measured in the intrinsic beat-to-beat variability case we used apex cell 1. The simulations had an initial cycle length of 230 ms, where alternans-like behaviour began to appear in the stochastic cell model using the apex cell 1 parameter set (Fig. 1B). The cycle length was reduced in 2 ms intervals after every 40 beats in the simulation until conduction block occurred. Conduction block was defined as a failure of the stimulus to lead to excitation of the entire tissue. Simulations were performed for both the deterministic and the stochastic models to determine the degree of BVR in tissue due to intrinsic variability.

#### 2.2.4 Comparing the relative effects of intrinsic and extrinsic variability

In order to simulate the effect of extrinsic inter-cell variability, one of the parameter sets corresponding to the four cells in Section 2.1 was applied to the cell model at each node in the computational mesh. The parameter sets were applied randomly with equal probability. Simulations were performed for both the deterministic (extrinsic variability only) and the stochastic version (extrinsic and intrinsic variability) of the resulting tissue model. 50 stimuli were applied at a pacing cycle length of 1000 ms. The same spatial distribution of cell parameter sets was used for both the deterministic and the stochastic simulation to enable direct comparison between results.

#### 2.2.5 Data analysis

Simulation results were analysed by recording APD_90_ after each simulation. We did not perform these analyses where x≤0.25cm or x≥0.75cm to prevent electrotonic effects arising from the Neumann boundary conditions or the stimulus site influencing the APD_90_ arising from the cell model (Cherry and Fenton, 2011). The maximum dispersion of APD was measured as the maximum difference in APD_90_ across the region 0.25 cm ≤x≤0.75cm at each node in the mesh. The difference in APD_90_ between the stochastic and the deterministic simulation was also computed for every stimulus. Furthermore, when considering BVR across multiple beats in the tissue, we calculated the mean APD_90_ in the tissue during each activation.

#### 2.2.6 Numerical implementation and simulation software

Simulations in tissue were performed using the finite element method, as implemented in the open source cardiac simulation package Chaste (Mirams et al., 2013). The deterministic or the stochastic cell model was applied to each node of the tissue mesh as appropriate. The solution of the deterministic ODE at each node in the mesh was determined using a forward Euler scheme, and the stochastic SDE models were solved using an Euler–Marayama scheme (Kloeden and Platen, 2011). Independent Weiner processes were generated for each node in the tissue and each stochastic variable at that node. Numerical convergence for both the deterministic and stochastic schemes was established and a spatial discretization of 0.01 cm, PDE time step of 0.01 ms, and an ODE/SDE time step of 0.00125 ms were used in this study, as described in Appendix B. A user project containing all of the code used in this study is available to download from the Chaste website (http://www.cs.ox.ac.uk/chaste).

## 3 Results

### 3.1 Intrinsic variability alone has a small effect on AP dispersion

**Fig. 2:**
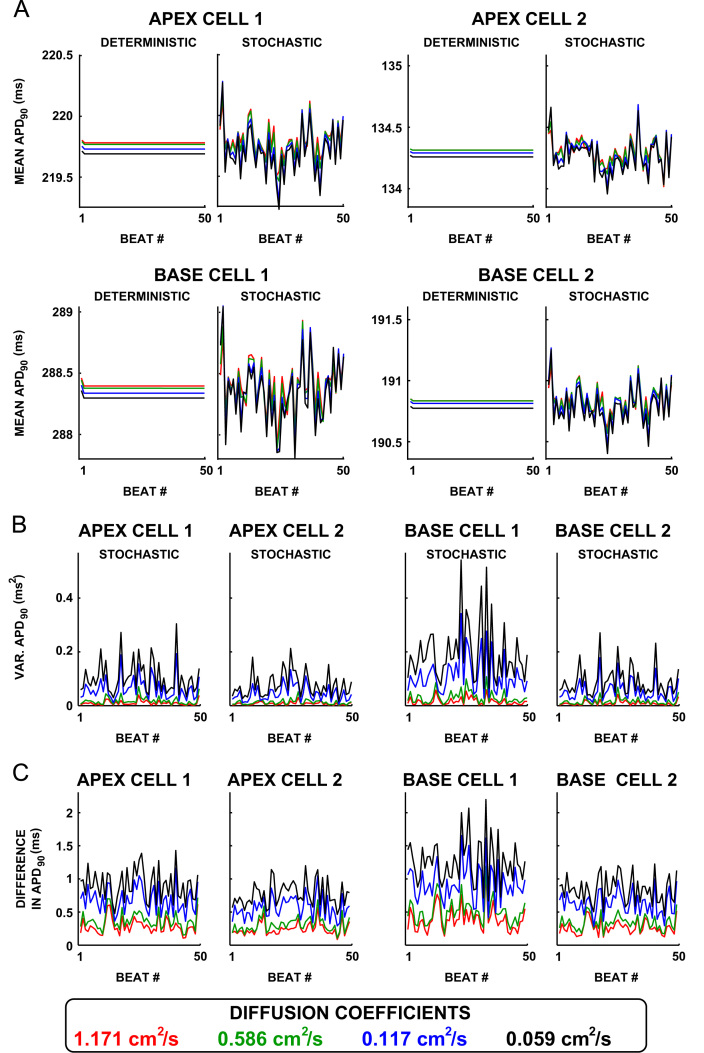
Reducing coupling in tissue simulations with intrinsic variability increases dispersion in APD_90_. (A) The mean APD_90_ across all nodes in the central 0.5 cm of the tissue is plotted for both the deterministic (left) and stochastic (right) simulations for each parameter set and each value of *D*. The values of *D* shown are 1.171 cm^2^ s^−1^ (red), *D*=0.586 cm^2^ s^−1^ (green), *D*= 0.117 cm^2^ s^−1^ (blue), and *D*= 0.059 cm^2^ s^−1^ (black). (B) The difference between the stochastic and deterministic APD_90_ maps is calculated at each node and each AP in the central 0.5 cm of the tissue. The variance across these nodes is then calculated for each beat. The variance in the difference between the deterministic and stochastic APD_90_ maps is plotted as a function of the number of beats. (C) The absolute difference between the deterministic and stochastic simulations is calculated at each node in the central 0.5 cm of the tissue. The maximum of these absolute differences for each beat are then plotted as a function of the number of beats. (For interpretation of the references to colour in this figure caption, the reader is referred to the web version of this paper.)

Tissue simulations of intrinsic variability only showed temporal variability in the mean repolarization time for each of the four parameter sets, shown in Fig. 2. Variability in mean APD_90_ between beats increased slightly as the diffusion coefficient *D* was decreased. This was quantified by calculating the variance in mean APD_90_ across all 50 beats (see Table 2). The distribution of mean APD_90_ for the stochastic simulations with intrinsic variability was centred on the mean APD_90_ of the deterministic simulations (no intrinsic variability) in all cases. The beat-to-beat variance in mean APD_90_ also increased with the variance in APD_90_ of the underlying cell (Table 2, see brackets after cell name for isolated cell variance in APD_90_).

**Table 2:** Summary of intrinsic variability only simulations $\mu_{\mathrm{APD}}$: the mean APD_90_ in the simulated tissue over one beat. Mean($\mu_{\mathrm{APD}}$): average of $\mu_{\mathrm{APD}}$ over all beats in the simulation. Var($\mu_{\mathrm{APD}}$): variance in $\mu_{\mathrm{APD}}$ over all beats in the simulation. **Det.**: deterministic simulations. Stoch.: stochastic simulations. Variance in brackets below each cell is from the isolated cell simulations shown in Fig. 1, shown for comparison.

Cell		*D* (cm^2^ s^−1^)			
		1.171	0.586	0.117	0.059
Apex cell 1	Det. Mean($\mu_{\mathrm{APD}}$) (ms)	219.8	219.8	219.7	219.7
(var. 39.22 ms^2^)	Stoch. Mean($\mu_{\mathrm{APD}}$) (ms)	219.8	219.8	219.7	219.7
	Stoch. Var($\mu_{\mathrm{APD}}$) (ms^2^)	0.020	0.023	0.033	0.037

Apex cell 2	Det. Mean($\mu_{\mathrm{APD}}$) (ms)	134.3	134.3	134.3	134.3
(var. 7.64 ms^2^)	Stoch. Mean($\mu_{\mathrm{APD}}$) (ms)	134.3	134.3	134.3	134.3
	Stoch. Var($\mu_{\mathrm{APD}}$) (ms^2^)	0.012	0.014	0.020	0.021

Base cell 1	Det. Mean($\mu_{\mathrm{APD}}$) (ms)	288.4	288.4	288.3	288.3
(var. 77.97 ms^2^)	Stoch. Mean($\mu_{\mathrm{APD}}$) (ms)	288.4	288.4	288.4	288.3
	Stoch. Var($\mu_{\mathrm{APD}}$) (ms^2^)	0.041	0.047	0.065	0.067

Base cell 2	Det. Mean($\mu_{\mathrm{APD}}$) (ms)	190.8	190.8	190.8	190.8
(var. 23.73 ms^2^)	Stoch. Mean($\mu_{\mathrm{APD}}$) (ms)	190.8	190.8	190.8	190.8
	Stoch. Var($\mu_{\mathrm{APD}}$) (ms^2^)	0.013	0.016	0.022	0.025

Spatial dispersion in the APD_90_ resulting from intrinsic variability, as measured by the variance in the difference between the stochastic and the deterministic simulation for each beat is shown in Fig. 2B. Fig. 2B shows that the variance increased as *D* decreased for each parameter set, demonstrating an increase in dispersion of APD_90_ resulting from intrinsic variability.

The difference between the deterministic and stochastic simulations did not increase with the number of beats, as shown in Fig. 2C, showing that intrinsic variability does not cause increased tissue level BVR over time in tissue. The maximum difference between the stochastic and deterministic simulations increased as the diffusion coefficient decreased. The largest difference observed was in base cell 1 with *D*= 0.059 cm^2^ s ^−1^, with a difference of 2.20 ms from the deterministic simulation.

**Fig. 3:**
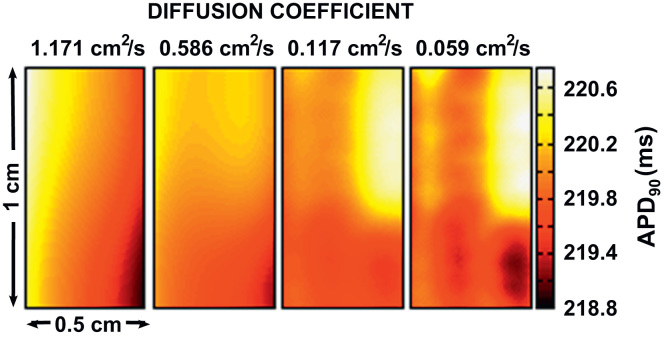
Heat maps of spatial dispersion in APD_90_ resulting from intrinsic variability for base cell 1. APD_90_ maps shown are recorded from base cell 1 with (A) *D* = 1.171 cm^2^ s^−1^, (B) *D* = 0.586 cm^2^ s^−1^, (C) *D* = 0.117 cm^2^ s^−1^, and (D) *D* = 0.059 cm^2^ s^−1^. The heat maps shown are generated using the stochastic cell model and are taken from the final beat in the series. APD_90_ maps are plotted from the central 0.5 cm to reduce distortion due to boundary effects.

Heat maps of the APD in the stochastic case for base cell 1 are shown in Fig. 3, demonstrating the increase in dispersion in APD resulting from intrinsic temporal variability in the cell model at low conductivities. A slight dispersion of APD arising from boundary effects can also be seen at higher conductivities, explained by the dependence of the size of the boundary effect with the conductivity of the tissue (Cherry and Fenton, 2011). This was also present in the deterministic simulations. The dispersion created a variance in both the deterministic and stochastic models at *D*= 1.171 cm^2^ s^−1^ of up to 0.18 ms^2^ in the deterministic case and 0.4 ms^2^ in the stochastic case (base cell 1).

### 3.2 Intrinsic variability can produce tissue level alternans at fast pacing rates

**Fig. 4:**
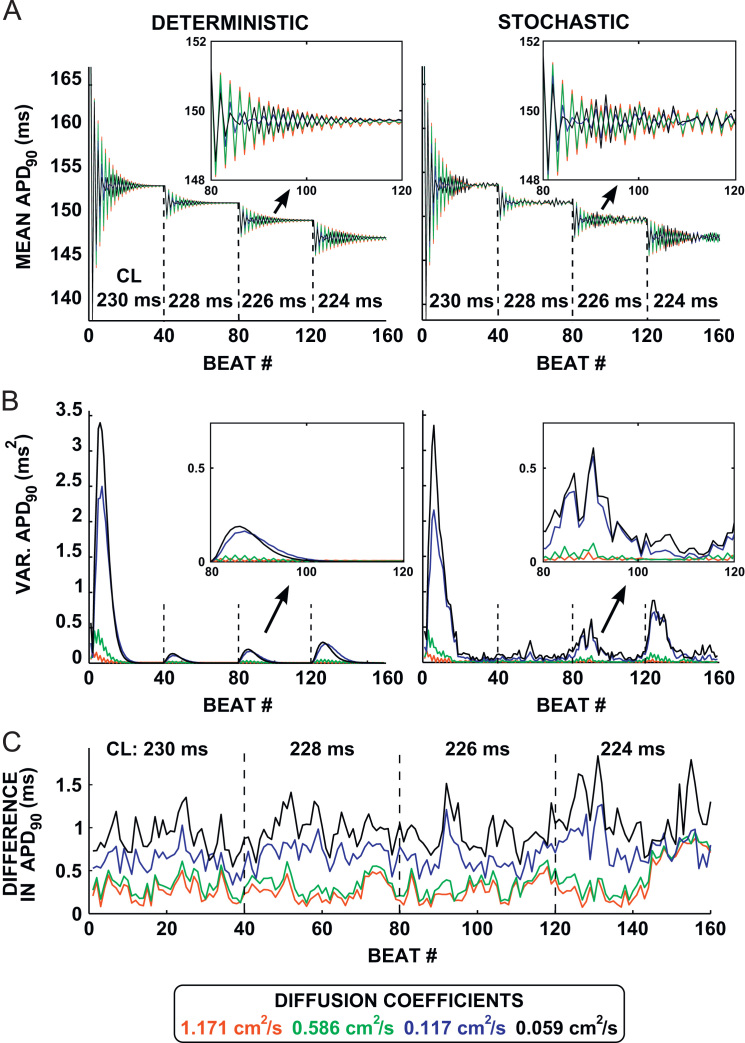
Summary statistics on maximum APD dispersion for the fast pacing protocol. Results are shown for *D*=1.171 cm^2^ s^−1^ (red), *D*= 0.586 cm^2^ s^−1^ (green), *D*= 0.117 cm^2^ s^−1^ (blue), and *D*= 0.059 cm^2^ s^−1^ (black). Vertical dashed lines denote a change in the pacing cycle length. (A) The mean APD_90_ is calculated across the nodes in the central 0.5 cm in the tissue at each beat in the deterministic (left) and stochastic (right) models. The variance of the APD is calculated over the nodes in the central 0.5 cm of the tissue for each beat. (B) The variance in APD_90_ is plotted for the deterministic and stochastic models. The insets show an enlarged view of beats 80–120. (C) The absolute difference in APD_90_ between the deterministic and stochastic simulations is calculated at each node in the central 0.5 cm of the tissue. The maximum absolute difference at each beat is shown. (For interpretation of the references to colour in this figure caption, the reader is referred to the web version of this paper.)

We ran simulations starting at a cycle length of 230 ms, and reduced the cycle length by 2 ms after every 40 beats until a conduction block occurred at a pacing cycle length of 222 ms. Fig. 4A shows a plot of mean APD_90_ recorded at all four values of *D* over 160 beats. Mean APD_90_ can be seen to oscillate dramatically in response to a change in cycle length, but oscillations are damped over time in both the deterministic and the stochastic cases, leaving a small beat-to-beat alternans in mean APD_90_.

We compute the variance within the APD_90_ map at each beat in order to evaluate the degree of dispersion in the APD, as shown in Fig. 4B. The dispersion is comparable between the stochastic and deterministic simulations following a change in pacing cycle length. In fact, the stochastic model displayed a reduced change in the mean value in the beats immediately following the change in cycle length as compared to the deterministic model, as is also seen in the isolated cell simulation in Fig. 1B. The transient increase in variance was due to the change in cycle length inducing a global alternans in both models that was modulated by boundary effects. To remove this effect, we also examined the mean and variance of the APD once the oscillations have stabilized in the final ten beats of the AP at each cycle length. For these beats, the mean value of the APD shows a moderately increased variability at low *D* as compared to the deterministic model. However, there is an increase in variance in the stochastic model, but not in the deterministic model, as the diffusion coefficient of the tissue is reduced, showing that the dispersion of the APD_90_ increases when including intrinsic variability at low values of *D*.

The overall effect of intrinsic variability on the simulation can be measured by calculating the maximum difference between the stochastic and deterministic simulations, as shown in Fig. 4C, for all values of *D*. The difference between the stochastic and deterministic simulations increases as *D* is reduced. The maximum difference for the lowest value of *D* (0.059 cm^2^ s^−1^) is bounded by ±1.5 ms, again smaller than the APD range in the stochastic version of apex cell 1 (31.4 ms).

**Fig. 5:**
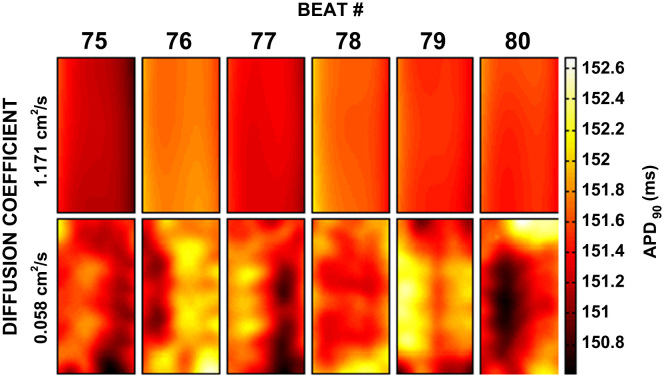
Spatially discordant alternans-like behaviour recorded in the action potential duration at 228 ms pacing cycle length in the stochastic model. The APD_90_ maps shown are simulated at a cycle length of 228 ms (beats 75–80) for the stochastic model for *D*= 1.171 cm^2^ s^−1^ and 0.059 cm^2^ s^−1^. APD_90_ maps are plotted from the central 0.5 cm only to remove boundary effects.

In the simulations with intrinsic variability, we observed phenomena that resembled a spatially discordant beat-to-beat alternans. Fig. 5 shows a sequence of six APD maps in the stochastic model from the end of the 228 ms pacing cycle length section of the pacing protocol at the lowest value of *D* (0.059 cm^2^ s^−1^). In the deterministic simulations, there is almost no spatial heterogeneity in the APD_90_, and similarly with *D*= 1.171 cm^2^ s^−1^ almost no dispersion is observed in the stochastic model. At *D*= 0.059 cm^2^ s^−1^, the stochastic simulations show a modest spatially heterogeneous alternation in the simulated APD_90_ that is not present in either the equivalent deterministic recording at the same value of *D*, or in either the stochastic or deterministic recording for *D*=1.171 cm^2^ s^−1^. The maximum amplitude of the oscillations is approximately 2 ms.

### 3.3 Extrinsic variability between cells has a greater effect on dispersion of repolarization than intrinsic variability in APD

**Fig. 6:**
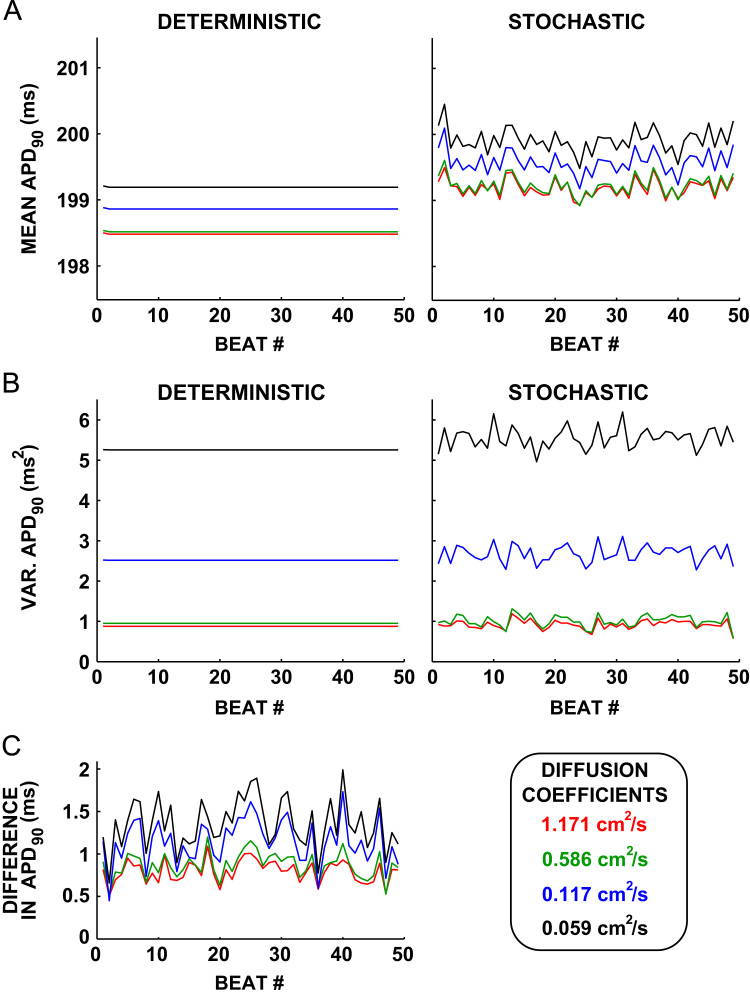
Mean and variance in APD_90_ from tissue simulations incorporating extrinsic variability. (A) The mean of the APD_90_ is calculated over all nodes in the central 0.5 cm of the tissue at each beat and plotted for both the deterministic simulations (left) and stochastic simulations (right) for each beat. (B) The variance in the APD_90_ for both the stochastic simulations (solid lines) and deterministic simulations (dashed lines) for each beat. (C) The maximum difference in APD between the stochastic and deterministic simulations at each beat, for each simulation. Values of *D* are 1.171 cm^2^ s^−1^ (red), *D*=0.586 cm^2^ s^−1^ (green), *D*= 0.117 cm^2^ s^−1^ (blue), and *D*= 0.059 cm^2^ s^−1^ (black). (For interpretation of the references to colour in this figure caption, the reader is referred to the web version of this paper.)

As in the intrinsic beat-to-beat variability-only simulations, the mean APD_90_ of the deterministic simulations was constant over all beats when including extrinsic variability between cells. There was a beat-to-beat variability in mean APD_90_ in the stochastic simulations that also included beat-to-beat intrinsic variability in the cells (Fig. 6A). Thus, given extrinsic variability in tissue generated from our four cells using a uniform random variable at each node, including intrinsic temporal variability, induces beat-to-beat variability in mean APD_90_ behaviour in tissue.

The variability in APD_90_ grows as *D* decreases due to the unmasking of extrinsic variability in the AP between cells, as shown by the increase in variance in the APD_90_ shown in Fig. 6B. The variance in the APD_90_ for the simulations of extrinsic variability between cells-only (deterministic) was slightly lower than when both extrinsic variability between cells and intrinsic variability from beat-to-beat in each cell were simulated (stochastic) as shown in Fig. 6B. However, there is still a much larger increase in the variance in the extrinsic variability-only case than is observed for any of the intrinsic variability only cases from Section 3.1. At the lowest value of *D*, the variance reaches a maximum of 6.1 ms^2^, which remains smaller than each simulated cell׳s variance in APD, which ranges between 17.64 ms^2^ for apex cell 2 and 77.97 ms^2^ for base cell 1 (Table 2). The difference between the deterministic and stochastic simulations remained at similar values to those seen for the intrinsic variability only simulations, being bounded between −1 ms and +2 ms at the lowest value of *D* (0.059 cm^2^ s^−1^). This is again dramatically smaller than the dispersion in the isolated cell simulations, which ranged between 22.3 ms for apex cell 2 and 47.1 ms for base cell 1.

**Fig. 7:**
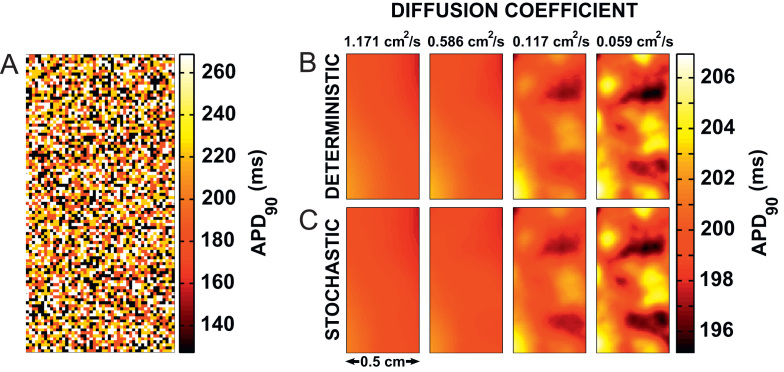
Distribution of APD_90_ from tissue simulations incorporating extrinsic variability. APD_90_ maps shown are recorded are for (A) the underlying cell distribution, then in (B) at *D*= 1.171 cm^2^ s^−1^, 0.586 cm^2^ s^−1^, 0.117 cm^2^ s^−1^, and 0.059 cm^2^ s^−1^ for the deterministic and stochastic simulation results. Simulation results shown are for the 50th beat in the series, and from the central 0.5 cm of the tissue only.

Heat maps of both deterministic and stochastic simulations from the final beat are shown in Fig. 7B, together with the underlying cell distribution in Fig. 7A. This figure demonstrates the increase in dispersion of repolarization as *D* is reduced. The maximum dispersion observed is 11 ms with *D*= 0.059 cm^2^ s^−1^. As the differences arising from the extrinsic variability between the simulated cells are much larger than those resulting from intrinsic temporal variability in the simulated cells, the heat maps in Fig. 7 are almost identical.

## 4 Discussion

In this study we have applied cell-specific parameterizations of a phenomenological AP model representing intrinsic temporal variability and extrinsic inter-cell variability to investigate consequences of variability in tissue. We have previously shown that a simple phenomenological model can reproduce cellular recordings of BVR (Walmsley et al., 2010). Using this model, we have now investigated the extent to which the intrinsic cellular APD impacts tissue level dispersion of APD, as well as the role of extrinsic cell–cell variability in APD. We have further investigated how this spatial dispersion in APD is affected by the coupling of the tissue.

In tissue simulations with intrinsic temporal variability only, we observe spatial dispersion of APD resulting from temporal variability in the APD of each of the four cells investigated. The dispersion of APD increases as the diffusion coefficient *D* is decreased, in accordance with previous studies. The dispersion resulting from temporal variability in the APD alone is relatively low. In absolute values, the maximum difference between simulations with and without intrinsic variability observed was around 2 ms in the case of base cell 1. We observed similar results at fast pacing rates, with the alternans observed both with and without intrinsic variability being damped in tissue. Intrinsic temporal variability in the APD produced a similarly low contribution to dispersion in the APD at fast pacing rates. BVR as observed in tissue is therefore unlikely to arise from cellular BVR alone. This does not exclude a role for ectopic activity such as early after depolarizations (Sato et al., 2009), or delayed after depolarizations (Johnson et al., 2013). We did not observe either of these phenomena in the four cells we investigated.

We additionally investigated whether the alternans-like behaviour observed in an intrinsic variability-only cell model at short pacing rates could cause increased dispersion of repolarization. Some simulations resemble a spatially discordant alternans in the simulations, which has been shown in animal models to be arrhythmogenic (Myles et al., 2008). The amplitude of this oscillation is small (2 ms) The amplitude of both the beat-to-beat and the spatially discordant alternans was dramatically lower than those in the single cell simulations (see Fig. 1B). One explanation for this phenomenon is that the stochastic nature of beat-to-beat variability in the model has resulted in some cells entering alternans with a long APD, and others entering alternans with a short APD, so that the overall behaviour in different regions is out of phase, but the difference is heavily damped by diffusion.

When including extrinsic variability between cells, we observed a spatial dispersion in APD that was visible even at the highest value of *D* (1.171 cm^2^ s^−1^). At the lowest value of *D* (0.059 cm^2^ s^−1^) this dispersion reached a value of 11 ms (Fig. 7), an order of magnitude larger than that observed in the temporal variability simulations in the intrinsic variability only cases (Fig. 3). This increase in dispersion resulting from inter-cell extrinsic variability alone can also be measured quantitatively by observing the large increase in variance in the extrinsic variability-only simulations as compared to the change in variance observed in the intrinsic variability-only simulations. Including intrinsic temporal variability in the extrinsic variability simulations resulted in a relatively small further increase in the dispersion as measured using variance. We conclude that the extrinsic cell-to-cell variability between the four cells we investigated provided a greater contribution to dispersion of repolarization in tissue than their intrinsic beat-to-beat variability.

Note that the degree of dispersion resulting from intrinsic beat-to-beat variability alone is too small to be detected even by microelectrode recordings. The dispersion observed in the signal when extrinsic variability is included is large enough that it could be detectable using microelectrode arrays. With a more spatially organized cell distribution, the dispersion, and hence its detectability, would increase; as seen in previous studies (Viswanathan et al., 1999).

When attempting to parameterize a model based on the AP morphology recorded from an individual cell, information on the conductances of all currents making up the observed AP are not available. Even if such information were available, ionic currents are modified by many factors, rendering them a ‘moving target’ (Roden, 2008, Carusi et al., 2012). Zaniboni et al. (2010) have shown that, due to poor parameter identifiability from the AP morphology alone, multiple behaviours in tissue are possible from apparently identical biophysically detailed models. Simplified phenomenological models such as the BOCF model therefore offer an alternative method for reproducing inter-cell variability based upon reproducing the APs recorded from individual cells (Fenton and Karma, 1998, Mitchell and Schaeffer, 2003, Bueno-Orovio et al., 2008, Bueno-Orovio et al., 2012). This method may avoid some of the risks associated with over-fitting biophysically detailed models to an individual AP, whilst still allowing investigation of the consequences of the observed behaviour. An alternative approach would be to use a calibrated population of biophysically detailed models based upon AP recordings from a large number of cells, as proposed by Britton et al. (2013), and then using the resulting parameter distributions to parameterize a tissue model containing extrinsic variability between cells.

Variability in resting potential was observed in the experiments (Fig. 1). As our work was focussed on intrinsic beat-to-beat variability, and the variability in the resting potential of these four cells was not correlated with their action potential durations, we did not include this effect. Future work using a modified phenomenological model, or a biophysically detailed model could consider the impact of this effect on dispersion of repolarization in tissue.

### 4.1 Limitations

The consequences of some cellular-level phenomena such as raised resting potential and accommodation of the AP in response to a change in pacing frequency cannot be investigated using the BOCF model. However, the more recent Bueno-Orovio et al. (2012) model does allow the inclusion of APD accommodation. In this study, our primary objective was to investigate the consequences of BVR in tissue. Therefore, we did not take into account mechanisms such as stochastic ion channel gating that may explain the source of fluctuations in membrane potential that lead to BVR. Exploring how stochastic gating phenomena contribute to BVR requires a biophysically detailed model for which a Markov formulation could be used within the framework of the Langevin equations (Dangerfield et al., 2012). We instead used a constant additive noise term within a phenomenological model as this approach was capable of reproducing the observed behaviour in a simple manner.

The nodes of a finite element mesh do not represent cells as such, but represent the homogenized behaviour of a small volume of cells. Thus, the level of extrinsic variability at each node will not correspond exactly to that in the isolated cells used for parameterization. Our intrinsic variability data arise from the number of ion channels (or, assuming uniform ion channel density, the membrane surface area) of a single cell. This may differ from the number of ion channels/surface area of membrane in the tissue volume represented by the AP model at a single mesh node, and so a future refinement of the variability to match mesh element size may be in order. At very low levels of cellular coupling, it may be necessary to use a discrete modelling approach as assumptions used in the monodomain model may break down. The approach presented here also inherits a limitation from the data set used for its parameterization. We only use four cells, and they come from different areas of two different hearts. Without further experiments we cannot say whether this variability arises as a result of abnormal cells, or in some way embodies the variability across one heart. Our conclusions are relevant to the coupling together of these four cells in tissue, rather than directly to the hearts in question.

In the real heart, the diffusion coefficient *D* is an anisotropic tensor whose principal direction follows the fibrous arrangement of myocytes within the myocardium (Hooks et al., 2007). In our simulations we have considered only isotropic diffusion coefficients. The interaction between fibre orientation and dispersion of repolarization arising from extrinsic and intrinsic variability may merit future study.

### 4.2 Conclusions

We have developed a phenomenological model capable of reproducing both intrinsic beat-to-beat variability in APD from an isolated guinea pig ventricular myocyte, and extrinsic variability in the AP between different cells. In this study, extrinsic and intrinsic variability were incorporated into tissue simulations. We conclude that, for the guinea pig APs used in this study, the degree of inter-cell variability makes a larger contribution to spatial dispersion in APD_90_ than beat-to-beat variability. The degree of dispersion in APD_90_ increases as tissue coupling is decreased. Furthermore, if BVR manifests in tissue, then it is likely to be as a result of ectopic activity, instead of the naturally occurring APD_90_ variability observed in isolated cardiac myocytes.

## Figures and Tables

**Fig. 8:**
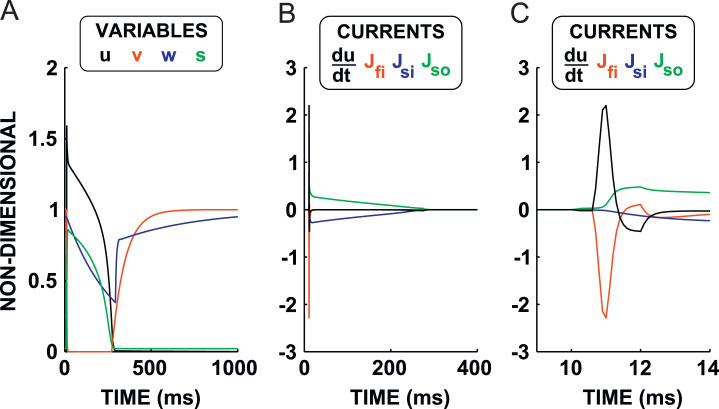
Variables and currents in the BOCF phenomenological model. The model is simulated using the human endocardial cell parameter set from Bueno-Orovio et al. (2008). (A) shows the behaviour of the four model variables *u* (black), *v* (red), *w* (blue), and *s* (green), when paced at a cycle length of 1000 ms. (B) and (C) show the behaviour of the total current (black), and the individual currents *J*_*fi*_ (red), *J*_*si*_ (blue), and *J*_*so*_ (green), when the cell is paced at 1000 ms. The time axes in (B) and (C) have been shortened to show structure of the currents, with (B) showing the whole AP and (C) showing the upstroke only. (For interpretation of the references to colour in this figure caption, the reader is referred to the web version of this paper.)

**Table A1:** Guinea pig cell-specific parameter sets for the BOCF model.

Parameter	Apex 1	Apex 2	Base 1	Base 2
*θ*_*v*_	0.2985	0.3209	0.2749	0.3005
*θ*_*w*_	0.1299	0.1302	0.1254	0.1202
$\theta_{v}^{-}$	0.2016	0.1943	0.1908	0.1714
*θ*_*o*_	0.006	0.0056	0.0058	0.0062
*u*_*s*_	0.9502	0.9802	0.9212	0.9391
$w_{w}^{-}$	0.77	0.77	0.77	0.77
*u*_*so*_	0.6456	0.6475	0.6645	0.7072
*u*_*u*_	1.546	1.617	1.592	1.575
*u*_*o*_	0.0001	−0.0008	0.0004	0.0003
$\tau_{w\infty }$	0.0273	0.041	0.036	0.029
*τ*_*o*1_	462.4	416.8	476.7	456.3
*τ*_*o*2_	5.951	6.1	5.579	6.042
*τ*_*s*1_	2.689	3.057	2.482	2.813
*τ*_*s*2_	1.977	2.192	1.764	1.949
*τ*_*so*1_	39.32	30.91	39.13	28.81
*τ*_*so*2_	1.193	0.2569	1.107	1.287
$\tau_{w1}^{-}$	80	80	80	80
$\tau_{w2}^{-}$	100	100	100	100
$\tau_{v1}^{-}$	75.39	76.26	72.99	66.59
$\tau_{v2}^{-}$	10.03	10.2	9.637	10.03
*τ*_*si*_	2.934	2.727	2.902	2.784
$\tau_{v}^{+}$	1.462	1.353	1.525	1.333
$\tau_{w}^{+}$	275.9	276.3	352.8	273.8
*τ*_*fi*_	0.0957	0.1094	0.095	0.0944
$w_{\infty }^{⁎}$	0.95	1	0.95	0.95
*k*_*so*_	1.946	2.006	2.023	1.942
$k_{w}^{-}$	198.2	204	188	207
*k*_*s*_	2.203	2.293	2.653	2.812
*θ*_*wo*_	1.1	1.1	1.1	1.1
*V* (mV)	94.1	103.8	94.6	90.5
*v*_*R*_ (mV)	−73.9	−77.8	−78.1	−76.1

**Fig. 9:**
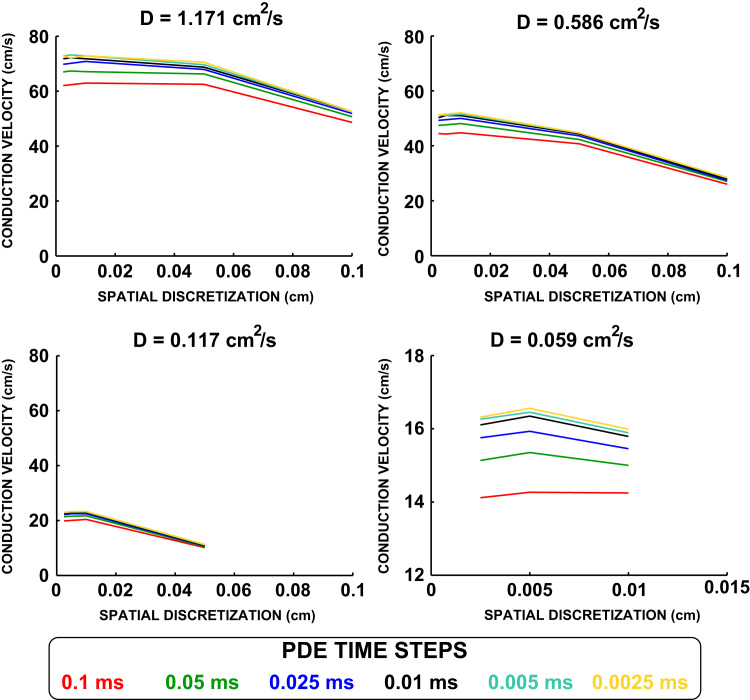
Convergence analysis for the deterministic model investigating the effects of PDE time step and spatial discretization for each value of D. Results are plotted for PDE time steps of 0.1 ms (red), 0.05 ms (green), 0.025 ms (blue), 0.01 ms (black), 0.005 ms (cyan) and 0.0025 ms (yellow). (A) shows results with *D*=1.171 cm^2^ s^−1^, (B) *D*= 0.586 cm^2^ s^−1^, (C) *D*= 0.117 cm^2^ s^−1^, and (D) *D*= 0.059 cm^2^ s^−1^. The axes on plot (D) have been rescaled to better display the results. The conduction velocity is not plotted where a propagation failure occurs. (For interpretation of the references to colour in this figure caption, the reader is referred to the web version of this paper.)

**Fig. 10:**
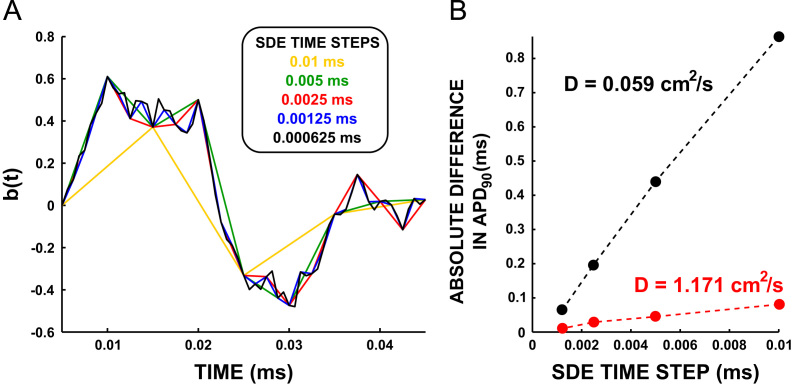
Convergence analysis for the stochastic model. (A) An example of a conserved Brownian path with nested paths at time steps of 0.01 ms (yellow), 0.005 ms (green), 0.0025 ms (red), 0.00125 ms (blue), and 0.00125 ms (black). (B) The absolute difference in APD_90_ is calculated across all nodes between the reference solution (timestep 0.000625 ms) and simulations at timesteps of 0.01 ms, 0.005 ms, 0.0025 ms, and 0.00125 ms. The maximum value of this difference is plotted for each time step with (A) *D*= 1.171 cm^2^ s^−1^ and (B) *D*= 0.059 cm^2^ s^−1^. (For interpretation of the references to colour in this figure caption, the reader is referred to the web version of this paper.)

## Appendix A The BOCF action potential model

The BOCF AP model has four variables, *u*, *v*, *w*, and *s. u* is the dimensionless membrane potential, rescaled to a biophysically relevant membrane potential using the formula $V_{m}=\mathrm{Vu}+V_{R}$, where *V* and *V*_*R*_ have dimensions of mV. *v*, *w*, and *s* are dimensionless variables whose decay controls the magnitude of the current. The model equations used in this study are as follows:

(A.1) $$\frac{\mathrm{du}}{\mathrm{dt}}=-(J_{\mathrm{fi}}+J_{\mathrm{so}}+J_{\mathrm{si}}-J_{\mathrm{stim}}),$$

(A.2) $$\frac{\mathrm{dv}}{\mathrm{dt}}=(1-H(u-\theta_{v}))(v_{\infty }-v)/\tau_{v}^{-}-H(u-\theta_{v})v/\tau_{v}^{+},$$

(A.3) $$\frac{\mathrm{dw}}{\mathrm{dt}}=(1-H(u-\theta_{w}))(w_{\infty }-w)/\tau_{w}^{-}-H(u-\theta_{w})w/\tau_{w}^{+},$$

(A.4) $$\frac{\mathrm{ds}}{\mathrm{dt}}=((1+\tanh(k_{s}(u-u_{s})))/2-s)/\tau_{s},$$

where *H* is the Heaviside function. The ‘currents’ $J_{\mathrm{fi}},J_{\mathrm{so}}$, and *J*_*si*_ are given as follows:

(A.5) $$J_{\mathrm{fi}}=-\mathrm{vH}(u-\theta_{v})(u-\theta_{v})(u_{u}-u)/\tau_{\mathrm{fi}},$$

(A.6) $$J_{\mathrm{so}}=(u-u_{o})(1-H(u-\theta_{w}))/\tau_{o}+H(u-\theta_{w})/\tau_{\mathrm{so}},$$

(A.7) $$J_{\mathrm{si}}=-H(u-\theta_{w})\mathrm{ws}/\tau_{\mathrm{si}},$$

and *J*_*stim*_ is the applied stimulus current. Furthermore, time constants depend on *u*:

(A.8) $$\tau_{v}^{-}=(1-H(u-\theta_{v}^{-}))\tau_{v1}^{-}+H(u-\theta_{v}^{-})\tau_{v2}^{-},$$

(A.9) $$\tau_{w}^{-}=\tau_{w1}^{-}+(\tau_{w2}^{-}-\tau_{w1}^{-})(1+\tanh(k_{w}^{-}(w-w_{w}^{-})))/2,$$

(A.10) $$\tau_{\mathrm{so}}=\tau_{\mathrm{so}1}+(\tau_{\mathrm{so}2}-\tau_{\mathrm{so}1})(1+\tanh(k_{\mathrm{so}}(u-u_{\mathrm{so}})))/2,$$

(A.11) $$\tau_{s}=(1-H(u-\theta_{w}))\tau_{s1}+H(u-\theta_{w})\tau_{s2},$$

(A.12) $$\tau_{o}=(1-H(u-\theta_{o}))\tau_{o1}+H(u-\theta_{o})\tau_{o2}.$$

The steady state values are given as

(A.13) $$v_{\infty }=\{\begin{array}{cc} 0, & u<\theta_{v}^{-},\\ 1, & u\geq \theta_{v}^{-}, \end{array}$$

(A.14) $$w_{\infty }=(1-H(u-\theta_{o}))(1-u/\tau_{w\infty })+H(u-\theta_{o})w_{\infty }^{⁎}.$$

The currents in the BOCF model represent an approximation to groups of currents observed in cardiac mycocytes. The fast inward current *J*_*fi*_ represents the sodium current *I*_Na_ that generates the upstroke of the AP. The plateau phase of the AP in the BOCF model is generated by a balance between two opposing currents. The slow inward current *J*_*si*_ represents inward currents such as the L-type calcium current *I*_CaL_, and the slow outward current *J*_*so*_ represents outward currents active in the plateau phase, such as the slow-rectifier and rapid-rectifier potassium currents *I*_Ks_ and *I*_Kr_. The variables *v*, *w*, and *s* represent the availability of channels generating these groups of currents to be activated. These variables recover following the end of the AP, as determined by the Heaviside functions in Eqs. (A.2), (A.3), (A.4). The Heaviside functions within the currents in Eqs. (A.5), (A.6), (A.7) approximate generic voltage-gated channel opening and closure. The values of the currents and variables making up the BOCF model during one AP are shown in Fig. 8. The parameter sets used in this study can be found in Table A1.

## Appendix B Convergence analysis for the stochastic tissue model

We examined convergence in the conduction velocity and in the maximum difference in the non-dimensional membrane potential *u* to determine the numerical parameters used in the tissue simulations using Eqs. (1), (2), (3), (4), as described in ‘Numerical implementation and simulation software’ in the Methods section.

Simulations are performed for each of the values of *D* in the main text. The spatial discretizations used are 0.1 cm, 0.05 cm, 0.01 cm, 0.005 cm, and 0.0025 cm. The PDE time steps used are 0.1 ms, 0.05 ms, 0.025 ms, 0.01 ms, 0.005, and 0.0025 ms. Deterministic convergence results are shown in Fig. 9. A spatial discretization of 0.01 cm and a PDE time step of 0.01 ms are sufficient for this simulation study.

We use a conserved Brownian path to investigate convergence properties of stochastic models (Gaines and Lyons, 1997). A conserved Brownian path is a path sampled such that at multiple temporal resolutions it remains a Brownian path. To construct such a path, we sample a Brownian path at our lowest temporal resolution of interest (0.01 ms). This Brownian path *b*(*t*) is sampled using a Weiner process whose Gaussian increment *W*_*h*_ has mean 0 and variance *h* on the increment [tt+h]. Hence $W_{t}\sim N(0,\sqrt{h})$. We now construct a new Brownian path from the original Brownian path, which preserves the increments of the old path, but has a temporal resolution of h/2. Consider each time increment [tt+h] in the original path in turn. We bisect this interval to get two subintervals [tt+h/2] and [t+h/2t+h]. We now wish to construct a random variable for each sub-increment, such that both new variables are Weiner increments on the subintervals [tt+h/2] and [t+h/2t+h]. Their sum must be equal to *dW*_*t*_, to conserve the original Brownian path.

Let $Z\sim N(0,\sqrt{h}/2)$ be independent of *W*_*h*_. Then consider the increments $\frac{1}{2}W_{h}-Z$ and $\frac{1}{2}W_{h}+Z$. These are then Weiner increments on the intervals [tt+Δt/2] and [t+Δt/2t+Δt], respectively. Using this method, we can iteratively construct a new Brownian path that preserves the previous Brownian path. This results in a nested series of Brownian paths, all with the same characteristics, at different temporal resolutions, as shown in Fig. 10A.

In order to test convergence we pre-generate a conserved Brownian path for the stochastic variables *w* and *s* for each node in a 0.5 cm × 0.5 cm tissue with a spatial discretization of 0.01 cm. Simulations are performed for a duration of 250 ms with the stochastic version of the apex cell 1 model. Simulations are analysed by calculating the difference in APD_90_ at each node between the current simulation and the reference solution obtained at a time step of 0.000625 ms (Fig. 10B). An absolute difference in APD_90_ of ≤0.1ms between the reference solution and the 0.00125 ms solution is observed in the *D*= 0.059 cm^2^ s^−1^ case. Simulations are run using a spatial discretization of 0.01 cm, a PDE time step of 0.01 ms and an ODE/SDE time step of 0.00125 ms.
